# Pet ownership is associated with harmful alcohol use among a cohort of people with HIV: a brief research report

**DOI:** 10.3389/fpsyt.2023.1258850

**Published:** 2023-10-16

**Authors:** Jennifer W. Applebaum, Shelby E. McDonald, Eric C. Porges, Maya Widmeyer, Humberto E. Fabelo, Darlene A. Kertes, Robert L. Cook

**Affiliations:** ^1^Department of Environmental and Global Health, University of Florida, Gainesville, FL, United States; ^2^Denver Zoological Foundation, Denver, CO, United States; ^3^Department of Clinical and Health Psychology, University of Florida, Gainesville, FL, United States; ^4^Unconditional Love, Inc., Melbourne, FL, United States; ^5^School of Social Work, Virginia Commonwealth University, Richmond, VA, United States; ^6^Department of Psychology, University of Florida, Gainesville, FL, United States; ^7^Department of Epidemiology, University of Florida, Gainesville, FL, United States

**Keywords:** pets, companion animals, people with HIV, alcohol use, substance use, HIV/AIDS, pet owners, human-animal interaction

## Abstract

Research suggests that people with HIV (PWH), who are at high risk for alcohol and substance use, may rely on relationships with pets for companionship and stress relief. There may be common mechanisms underlying both substance use and attachment to pets. The purpose of this brief research report was to compare alcohol and substance use behaviors between pet owners and non-owners among a cohort of PWH. Participants (*n* = 735) in a survey study of PWH in Florida were asked about their alcohol and substance use behaviors, whether they owned a pet, and their sociodemographic characteristics. We used bivariate analyses and logistic regression to examine differences in alcohol and substance use behaviors between pet owners and non-owners. Pet owners had higher mean AUDIT scores than non-owners (*M*_pet_ = 5, *M*_nopet_ = 4, z = −3.07, *p* = 0.002). Pet owners were more likely than non-owners to use alcohol in a harmful or hazardous way (AUDIT score ≥ 8), above and beyond sociodemographic characteristics (*OR* = 1.65, *p* = 0.052). Pet owners were more likely to have ever used most substances than non-owners, and more likely to currently use alcohol (*X*^2^(1) = 12.97, *p* = 0.000), marijuana or hashish (*X*^2^(1) = 6.82, *p* = 0.009), and amyl nitrate/poppers (*X*^2^(1) = 11.18, *p* = 0.001). Pet owners may be more likely to use alcohol and other substances at higher rates than non-owners. Reasons for owning a pet and using substances may be similar, such as coping with stress.

## 1. Introduction

Alcohol and substance use are highly prevalent among people with HIV (PWH) and are determinants of medication adherence and secondary transmission risk (1, 2). Identifying psychosocial correlates of alcohol and substance use among PWH is of critical importance both for public health and individual prognosis (1, 3, 4). Although a majority of U.S. households have at least one pet in the home (5–7), no prior studies to our knowledge have examined pet ownership as a potential psychosocial correlate of alcohol and substance use among PWH, despite research linking pet ownership with a variety of human health outcomes (6, 8–11). It is unclear whether pet ownership and substance use may be correlated in PWH or in what direction, owing to the complex physical, social, and economic challenges of living with HIV.

One possibility is that pet ownership may be linked with lower rates of alcohol and substance use in this population. There is some evidence that pets serve as meaningful relationships for PWH. Specifically, previous studies on pet ownership among PWH suggest that for some, pets offer companionship and a nonjudgmental source of emotional support (12–18). Moreover, caring for pets can provide a meaningful social role that promotes effective self-management of HIV via stress reduction and daily caregiving tasks (18). Thus, it is possible that pet ownership may be associated with lower alcohol and substance use among PWH by providing opportunities to engage in positive coping behaviors.

Alternatively, it is also plausible that pet ownership is associated with higher rates of alcohol and substance use among PWH. At a socioemotional level, people may engage in substance use for coping with distressing emotional states and symptoms (e.g., depression, internalized HIV stigma/self-stigma) social enhancement and intoxication (i.e., to feel good and fit in), and improvement of internal emotional and physical states (19–21). Research suggests that people seek out pet ownership for similar reasons: to foster relationships, experience companionship, and to provide stress relief and psychological benefits (22, 23). At a biological level, neuromodulators that impact responses to stressful experiences as well as social affiliative behavior such as OXTR are implicated in both alcohol misuse and interactions with companion animals. For example, OXTR rs53576 is recognized as a potential genetic locus for sensitivity to the social environment, and A-carriers at this locus have been reported to engage in more substance use among males (24, 25) and engage in more petting behavior during human-animal interaction (26). For PWH, a pet may provide social and emotional comfort, but may not fully alleviate anxiety and stress. Therefore, it is possible that motivations to manage negative affectivity and cope with stress may contribute to high degrees of overlap between pet ownership and substance use.

Pets (i.e., dogs) can also act as social lubricants (27); accordingly, pet ownership may increase opportunities for social engagement, and while reduced perceived social isolation has been associated with negative mental health outcomes (e.g., anxiety and depression), alcohol and other substances of abuse are often used in social situations and may also be impacted by this same mechanism. Finally, due to the level of responsibility required to adequately care for pets, as well as associated economic stressors (e.g., veterinary care), PWH who own pets may experience a higher level of stress than non-owners, which could contribute to higher rates of negative coping strategies such as alcohol and substance use in this population (28).

Given that pet owners are systematically different from non-pet owners with regard to several demographic and contextual characteristics (e.g., gender, race, income), it is important that research accounts for these factors when comparing health outcomes for PWH and how they may vary by pet ownership status (6, 29, 30). The purpose of this brief research report was to report comparisons in alcohol and substance use behaviors between pet owners and non-owners among a cohort of PWH while adjusting for the potential confounding effects of sociodemographic characteristics.

## 2. Methods

Data were from Wave 3 of the Florida Cohort, a survey of PWH in Florida, run by the Southern HIV and Alcohol Research Consortium (SHARC) in 2021–2023. Wave 2 of the Florida Cohort is described in Ibanez et al. (31) and had a similar methodology and goals to Wave 3. Participants were recruited at HIV care providers, patient registries, participant referrals, and remotely via advertising. Participants completed several survey modules, available in English, Spanish, and Haitian Creole, regarding general health, health care utilization, behavioral and social factors, alcohol and substance use, mental health, and pet ownership. Participants were compensated for each module they completed. The study was approved by the University of Florida Internal Review Board.

### 2.1. Measures

Alcohol use: The Alcohol Use Disorders Identification Test (AUDIT) is a validated 10-item questionnaire measuring alcohol consumption, drinking behavior, and alcohol-related problems (32). Possible scores on the AUDIT range from 0–40; based on AUDIT scoring criteria, participants who scored eight or above were coded as using alcohol in a harmful or hazardous way.

Other substances: Participants were asked if they had ever used several substances, including tobacco, marijuana, heroin, cocaine, stimulants, MDMA, opioids, hallucinogens, and amyl nitrate. Those who endorsed ever having used each substance were asked if they had used the substance in the past 12 months.

Pet ownership: Participants were asked, “do you have any pets?” Possible responses were “yes” or “no.”

Sociodemographic characteristics: Participants self-reported their age, race, Hispanic ethnicity, yearly family income, educational attainment, gender, and marital or relationship status.

### 2.2. Analytic procedures

We present bivariate analyses (Wilcoxon signed rank tests, chi-squared tests, and *t*-tests) to compare alcohol and substance use behaviors between pet owners and non-owners. We estimate a multivariate logistic regression to isolate the association between pet ownership and harmful or hazardous alcohol use above and beyond sociodemographic characteristics. We control for characteristics with known associations to alcohol use behaviors and/or pet ownership.

Of the 735 total participants, 546 both completed the AUDIT and had complete information on all variables of interest and are thus included in the multivariate analyses. Bivariate analyses and descriptive information are included for all non-missing observations.

## 3. Results

### 3.1. Descriptive information

Among the 735 Florida Cohort participants, 43% were pet owners. Participants were aged 20–80 years; the mean age for pet owners was slightly younger (*M* = 48, SD = 12.5) than non-owners (*M* = 51, SD = 13.6; *t* (733) = 2.93, *p* = 0.004). Race, Hispanic ethnicity, income, education, and marital status varied significantly between pet owners and non-owners. Sixty-two percent of pet owners identified their race as White, 33% Black, and 5% other races,^1^ while 21% of non-owners identified as White, 70% as Black, and 9% as other races (*X*^2^ (2)=124.46, *p* = 0.000). Twenty-one percent of pet owners endorsed Hispanic ethnicity, versus 12% of non-owners (*X*^2^ (1)=12.53, *p* = 0.000). Among pet owners, 26% made less than $10,000 per year, 38% made $10,000-29,999, 22% made $30,000-49,999, and 14% made $50,000 and above; among non-owners, 44% made less than $10,000, 35% made $10,000-29,999, 13% made $30,000-49,999, and 8% made $50,000 and above (*X*^2^ (3)=31.03, *p* = 0.000). Sixteen percent of pet owners reported less than a high school education, 32% reported high school or GED attainment, and 52% reported some college and above; 32% of non-owners had less than high school education, 27% had high school or GED, and 41% reported some college and above (*X*^2^ (2)=24.76, *p* = 0.000). Among pet owners, 48% were single, 9% were living with a long-term partner, 16% were married, and 26% were divorced, separated, or widowed; among those who did not own pets, 56% were single, 4% were living with a long-term partner, 12% were married, and 28% were divorced, separated, or widowed (*X*^2^ (3)=11.92, *p* = 0.008). Gender did not vary significantly between pet owners and non-owners: 60% of pet owners were male versus 55% of non-owners, 38% of pet owners were female, versus 43% of non-owners, and 3% of pet owners reported other genders, versus 1% of non-owners (*X*^2^ (2)=3.87, *p* = 0.144).

### 3.2. Differences in alcohol and substance use behaviors between pet owners and non-owners

Pet owners had significantly higher AUDIT scores and were more likely to use alcohol in a hazardous or harmful manner (≥8 AUDIT score) than non-owners. Pet owners were significantly more likely to have ever used alcohol, cigarettes, marijuana or hashish, stimulants, unprescribed opioids, ecstasy or MDMA, hallucinogens, and amyl nitrate or poppers than non-owners. In the past 12 months, pet owners were more likely than non-owners to have used alcohol, marijuana or hashish, and amyl nitrate. See Table 1 for statistical information.

**Table 1:** Pet owners versus non-owners: alcohol and substance use behaviors.

Variable	Pet	No pet	*p*-value	*X*^2^(df)/z	*n* ^a^
AUDIT score in past year (0–39)^**^	5 (*M*) (*SD* = 6.9)	4 (*M*) (*SD* = 6.7)	0.002	−3.07	566
Hazardous alcohol use in past year^*^	25%	18%	0.042	4.14 (1)	566
*Ever used:*					
Alcohol^***^	91%	81%	0.000	14.32 (1)	731
Cigarettes^**^	70%	60%	0.004	8.14 (1)	731
Marijuana or hashish^**^	75%	64%	0.001	10.30 (1)	733
Heroin (snort or smoke)	10%	11%	0.799	0.06 (1)	733
Injection drugs	14%	14%	0.931	0.01 (1)	733
Cocaine or crack	48%	43%	0.206	1.59 (1)	733
Stimulants^***^	28%	17%	0.000	15.07 (1)	733
Opioids (not as prescribed)^***^	22%	12%	0.000	14.29 (1)	733
Ecstasy/MDMA^*^	22%	15%	0.014	6.01 (1)	733
Hallucinogens^**^	23%	14%	0.001	10.96 (1)	733
Amyl nitrate/poppers^***^	33%	16%	0.000	30.43 (1)	733
*Used in past year:*					
Alcohol***	73%	60%	0.000	12.97 (1)	731
Cigarettes	37%	34%	0.363	0.83 (1)	731
Marijuana or hashish^**^	46%	36%	0.009	6.82 (1)	725
Heroin (snort or smoke)	1%	2%	0.368	0.81 (1)	730
Injection drugs	4%	5%	0.423	0.64 (1)	733
Cocaine or crack	9%	12%	0.177	1.82 (1)	723
Stimulants	9%	6%	0.238	1.39 (1)	729
Opioids (not as prescribed)	6%	3%	0.125	2.36 (1)	733
Ecstasy/MDMA	5%	4%	0.431	0.62 (1)	732
Hallucinogens	3%	2%	0.380	0.77 (1)	732
Amyl nitrate/poppers^**^	16%	8%	0.001	11.18 (1)	733

a Sample sizes vary due to skip logic, module design, and missing observations.

^***^p < 0.001, ^**^p < 0.01, ^*^p < 0.05.

**Figure 1:**
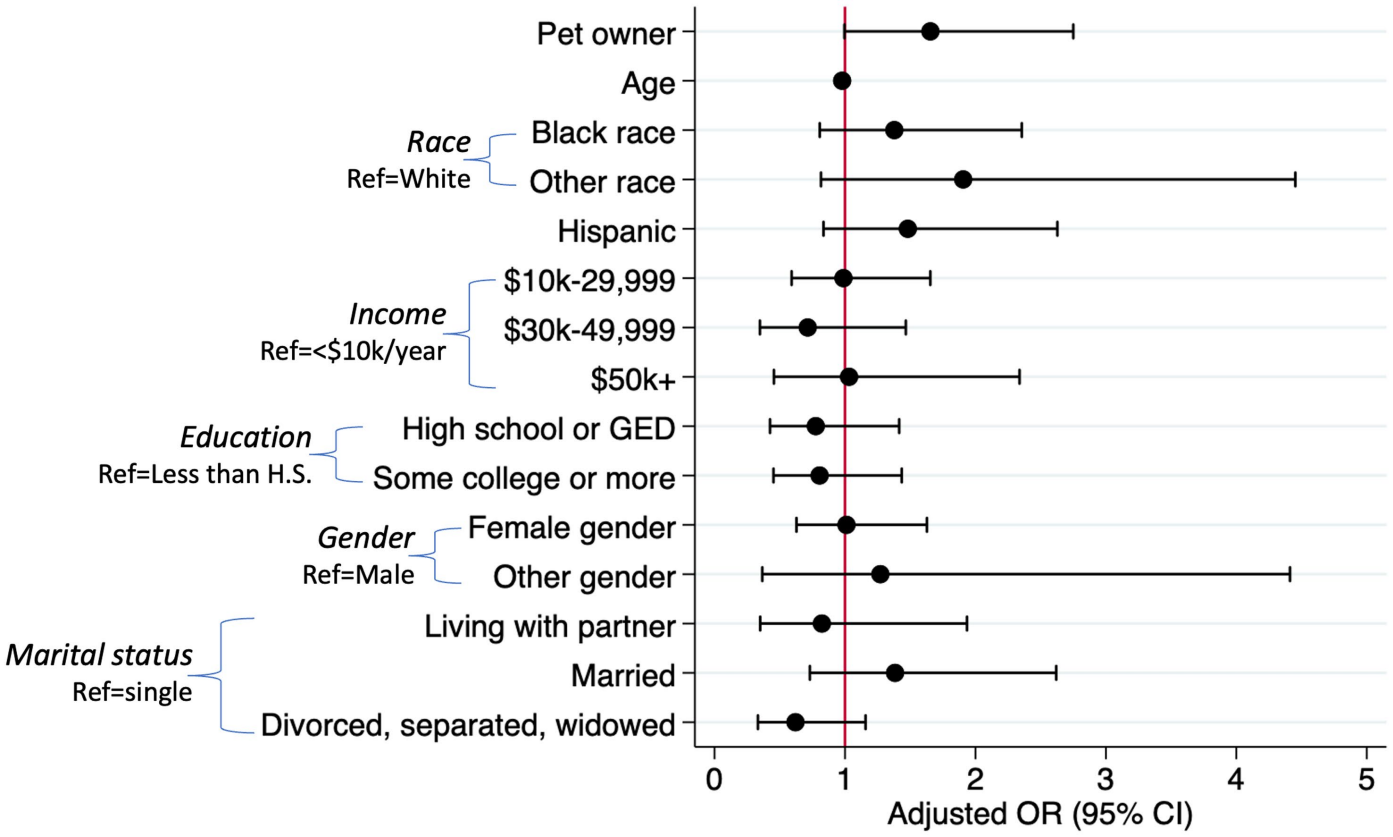
Logistic regression predicting harmful alcohol use (AUDIT score of 8+) (*n* = 546).

In the logistic regression model (LR *X*^2^ (15)=29.44, *p* = 0.014), pet ownership was marginally significantly associated with harmful or hazardous alcohol use (≥8 AUDIT score) above and beyond the effects of age, race, Hispanic ethnicity, income, education, gender, and marital status (OR = 1.65, *p* = 0.052). Age was the only other significant variable in the model: older participants were less likely to be harmful or hazardous alcohol users than younger participants (OR = 0.98, *p* = 0.015). Figure 1 displays the odds ratios and confidence intervals for each variable in the model.

## 4. Discussion

In this brief research report, we described differences in alcohol and substance use behaviors between pet owners and non-owners among a cohort of PWH. We found that pet owners were more likely to be current alcohol users than non-owners, tended to score higher on a standard assessment of harmful or hazardous alcohol use, and this association remained marginally statistically significant when adjusting for the effects of sociodemographic covariates in the model. Additionally, pet owners were more likely to have ever used most substances than non-owners and more likely to be current users of marijuana or hashish, and amyl nitrate.

The higher likelihood of harmful alcohol use among pet owners (compared to non-owners) in this study, at a surface level, may be somewhat counter-intuitive in the context of previous research among this population suggesting that pets may contribute to well-being and motivate PWH to maintain healthy lifestyles (13–16, 18). However, some research among PWH and other populations has shown that pet owners with strong attachment bonds may have poorer mental health than those with weaker bonds (9, 13, 34), suggesting that reliance on a pet for emotional support could be indicative of a greater need for coping mechanisms. Additionally, this study is cross-sectional and did not account for the participants’ duration of pet ownership, years living with HIV, and duration of alcohol and substance use; other research has suggested that the mental health effects of pet ownership could emerge over time (35). Future research should account for these factors.

If both pet ownership (i.e., attachment to pets) and substance and alcohol use serve similar coping roles, it may be possible to harness the human-animal bond to reduce the harmful or hazardous use of alcohol and other substances. For example, if a pet owner uses substances to relieve stress, creating interventions to strengthen the human-animal bond (e.g., engaging in activities that are mutually beneficial for both human and pet) may help to reduce substance use behaviors via pet-associated stress reduction. Importantly, broader public and community-based support for pet ownership among marginalized populations, such as PWH, is pertinent in order to relieve some of the stress of pet caregiving and thus encourage healthy coping via pet support, versus unhealthy coping with substances. Pet ownership, without broader support for the human-animal bond, may serve to create more stress and thus encourage substance use, rather than reduce it. Future research should assess whether programs to support pet ownership among PWH may encourage healthy coping and thus reduce alcohol and substance use.

Though attachment to pets was not assessed in this study, previous research suggests there are common biological, psychological, and social mechanisms underlying both heavy alcohol use and strong attachments to pets. For example, recent experiences of stress, state or trait anxiety, and adverse childhood experiences coupled with neurobiological predispositions to experience altered responses to stress and social interaction including but not limited to the oxytocinergic system may all play common roles in both substance use and attachment to pets (36). These factors, both individually and in combination, should be assessed in future research on pet ownership and alcohol and substance use, particularly among PWH and other marginalized populations.

## Data availability statement

The datasets for this article are not publicly available due to concerns regarding participant/patient anonymity. Requests to access the datasets should be directed to the corresponding author.

## Author contributions

JA: Conceptualization, Formal analysis, Funding acquisition, Investigation, Methodology, Software, Visualization, Writing – original draft, Writing – review & editing. SM: Conceptualization, Funding acquisition, Writing – original draft, Writing – review & editing. EP: Conceptualization, Writing – review & editing. MW: Data curation, Investigation, Writing – review & editing. HF: Data curation, Writing – review & editing. DK: Writing – review & editing. RC: Conceptualization, Funding acquisition, Investigation, Resources, Supervision, Writing – review & editing.

## Footnotes

^1^This approach was employed for analytic purposes, as small group sizes are not compatible with the methodologies used in this paper. There are limitations to grouping racial and ethnic groups; for a discussion of the issues this approach can raise see (33).
